# Asthma from Suppressed Eruption

**Published:** 1896-05

**Authors:** 


### ASTHMA FROM A SUPPRESSED ERUPTION.

DR. DAHLKE.

(Translated by A. McNeil, M. D., San Francisco, Cal., from *Die Zeitschrift.*)

R., a teacher, about forty, came to me in the summer of 1892 on account of asthma, from which he had suffered three years. He thinks it has been caused by taking cold. The attacks usually arise after catching cold, and continue several days at least. They begin with whistling in the chest, and dyspnœa when walking. This increases to such a degree that the slightest motion produces a want of breath. The first days there is neither expectoration nor cough afterward. This is followed by expectoration of mucous threads, without cough. When the attacks are severe he cannot go to bed, and must sit bent forward. Fumigation with Stramonium and Hyoscyamus relieves.

He had suffered since his sixteenth year from an eruption which disappeared with the beginning of the asthma, and has not returned.

During winter he suffers most. He is a demi-blonde, inclined to corpulence. I gave him Sulphur $^{5}$, which he took until late in autumn.

January 27th, 1893.—He has been all winter, until the present time, free from a severe attack. The eruption has returned and troubles him much with itching.

Sulphur $^{30}$, a dose night and morning for three days, then Placebo.

March 6th.—He has not had an attack. Continue Placebos.

February 8th, 1894.—Has not had an attack this winter.

---

EXANTHEMA SQUAMOSUM FROM QUININE.—Of all acute exanthemata none is followed by such thorough desquamation as scarlatina and the Quinine eruption. All medicinal eruptions have this in common, that they are very apt to return after every new use of the drug.

